# Immunoassays for the quantification of ALK and phosphorylated ALK support the evaluation of on‐target ALK inhibitors in neuroblastoma

**DOI:** 10.1002/1878-0261.12069

**Published:** 2017-05-31

**Authors:** Elizabeth R. Tucker, Jennifer R. Tall, Laura S. Danielson, Sharon Gowan, Yann Jamin, Simon P. Robinson, Udai Banerji, Louis Chesler

**Affiliations:** ^1^ Paediatric Solid Tumour Biology and Therapeutics Team Division of Clinical Studies and Cancer Therapeutics Division The Institute of Cancer Research Sutton Surrey UK; ^2^ Clinical Pharmacodynamic Biomarker Team Cancer Therapeutics Division The Institute of Cancer Research Sutton Surrey UK; ^3^ Tumour Biology and Metastasis Cancer Therapeutics Division The Institute of Cancer Research Sutton Surrey UK; ^4^ Division of Radiotherapy and Imaging The Institute of Cancer Research Sutton Surrey UK

**Keywords:** ALK, neuroblastoma

## Abstract

Targeted inhibition of anaplastic lymphoma kinase (ALK) is a successful approach for the treatment of many ALK‐aberrant malignancies; however, the presence of resistant mutations necessitates both the development of more potent compounds and pharmacodynamic methods with which to determine their efficacy. We describe immunoassays designed to quantitate phosphorylation of ALK, and their use in preclinical models of neuroblastoma, a pediatric malignancy in which gain‐of‐function ALK mutations predict a poor overall outcome to conventional treatment. Validation of the immunoassays is presented using a panel of neuroblastoma cell lines and evidence of on‐target ALK inhibition provided by treatment of a genetically engineered murine model of neuroblastoma with two clinical ALK inhibitors, crizotinib and ceritinib, highlighting the superior efficacy of ceritinib.

**Abbreviations**
ALK: anaplastic lymphoma kinase
MSD^®^: Meso Scale Discovery

## 1 Introduction

The rapid development of targeted therapeutics against anaplastic lymphoma kinase (ALK) has already resulted in significant changes to the up‐front treatment of patients with ALK‐rearranged non‐small‐cell lung cancer (NSCLC) (Camidge *et al*., 2012), and a current aim is to bring these personalized therapies to the benefit of childhood cancer patients, including those with neuroblastomas that harbor point mutations of ALK (Chen *et al*., 2008; George *et al*., 2008; Janoueix‐Lerosey *et al*., 2008; Mosse *et al*., 2008). The broad range of ALK mutations found either as a secondary resistance mechanism to ALK inhibition in NSCLC or as a primary resistance mechanism in neuroblastoma necessitates thorough interrogation of the ability of individual compounds to inhibit ALK phosphorylation and subsequent survival signaling downstream of ALK, enabling the rapid translation of the most promising compounds from the laboratory to clinical trials (Bresler *et al*., 2011, 2014). To achieve this, highly relevant preclinical models should be coupled with accurate methods to report pharmacodynamic responses.

We have therefore developed sensitive immunoassays to detect and quantitate the expression of total ALK protein and phosphorylated forms of ALK using the mesoscale technology (MSD^®^) platform. This format allows for the development of assays that are appropriate for both *in vitro* and *in vivo* studies, and MSD^®^ assays are already routinely incorporated into clinical studies in order to measure pharmacodynamic end points (Basu *et al*., 2015). We sought to validate our ALK immunoassays using both neuroblastoma cell lines and tumor tissue from the Th‐*ALK* ^*F1174L*^/*MYCN* transgenic model, which has previously demonstrated the inadequacy of the first‐generation ALK inhibitor, crizotinib, to elicit therapeutic responses in ALK F1174L‐driven neuroblastomas (Berry *et al*., 2012).

Our results demonstrate the successful application of MSD^®^ immunoassays to measure ALK and phosphorylated ALK as pharmacodynamic biomarkers following treatment with small‐molecule ALK inhibitors in both *in vitro* and *ex vivo* tissues. We show that in addition to autophosphorylation of ALK at Y1278 and Y1604, following the fate of phosphorylated ALK at Y1586 also provides a marker of active ALK levels.

## 2 Materials and methods

### 2.1 Cell lines

Neuroblastoma cell lines and HeLa cells were obtained from the American Type Culture Collection, CLB‐GA was a gift from V. Combaret (Lyon), and these were shown to be mycoplasma‐free using a PCR‐based assay (Minerva Biolabs, Berlin, Germany). The Ba/F3 ALK F1174L cells were a gift from R. George (Boston, USA) and were transduced as described previously (George *et al*., 2008). Cells were cultured in RPMI 1640 media supplemented with 2 mm glutamine (Gibco, Thermo Fisher Scientific, Waltham, MA, USA), 1 × MEM nonessential amino acids, and 10% FBS (Gibco) and grown at 37 °C with 5% CO_2_ in a humidified incubator.

### 2.2 Drug treatments

Cell lines were treated at 50–70% confluency at the indicated drug concentrations or with DMSO at a concentration that matched the greatest DMSO for the drug‐treated cells. Crizotinib and ceritinib were purchased from Shanghai Haoyuan Chemexpress Company.

### 2.3 GI_50_ determination

In order to calculate the half maximal growth inhibitory concentration (GI_50_) of individual compounds, neuroblastoma tumor cells were seeded into 96‐well plates in a total volume of 100 μL and allowed to attach overnight. Compound (dissolved in DMSO) was added to wells in six replicates of 100 μL, across a concentration gradient including a DMSO‐only control, the next day. The cells were exposed to drug for 72 h. Thereafter, the cell number in treated versus control wells was estimated after cell fixation with 10% trichloroacetic acid and staining with sulforhodamine B in 1% acetic acid. The GI_50_ was calculated as the drug concentration that inhibits cell growth by 50% compared with control growth, according to nonlinear regression analysis, using graphpad prism (La Jolla, CA, USA).

### 2.4 Preparation of protein lysates

Cell lines were harvesting by scraping, spun at 500 *g* for 5 min, and washed once in phosphate‐buffered saline, and the cell pellets were resuspended in CHAPS lysis buffer [50 mm Tris/HCl pH 8.0, 1 mm EDTA, 150 mm NaCl, 1% CHAPS, 0.2 mm PMSF, 1 : 50 Phosphatase Inhibitor Cocktail 2 and 3 (Sigma‐Aldrich, St. Louis, MO, USA), 1 : 100 Protease Inhibitor Cocktail (Sigma‐Aldrich)]. Frozen tissue samples were homogenized in CHAPS lysis buffer prepared as for cell lysates. After incubation for 30 min on ice, lysates were spun at 16 000 ***g*** for 15 min and the supernatant was collected. Protein concentrations were determined using BCA protein assay (Thermo Fisher Scientific) by comparison with bovine serine albumin standard.

### 2.5 ALK Meso Scale Discovery^®^ immunoassays

Multiarray 96‐well plates (Meso Scale Discovery) were coated overnight at 4 °C with 0.5 μg·mL^−1^ mouse total ALK antibody (Clone 31F12; Cell Signaling Technology Inc., Danvers, MA, USA) diluted in 50 mm carbonate buffer. Plates were washed 5 × in wash buffer (0.1% Tween 20 in Tris‐buffered saline) and incubated for 1 h with blocking buffer (5% BSA in wash buffer). After washing, samples were added with cell lysates being diluted to 20 μg per well (*in vitro*) or 30 μg per well (*in vivo*) in 1 × Tris Lysis Buffer (Meso Scale Discovery) or recombinant ALK protein (Thermo Fisher Scientific) diluted in phosphate‐buffered saline and incubated overnight at 4 °C. After washing, the plates were then incubated for 1 h with the appropriate antibody (0.4 μg·mL^−1^ total ALK D5F3, 0.2 μg·mL^−1^ pY1278 ALK D59G10, 0.4 μg·mL^−1^ pY1586 ALK 3B4, 0.2 μg·mL^−1^ pY1604 ALK D96H9, or 0.2 μg·mL^−1^ phospho‐tyrosine P‐Tyr‐1000, all purchased from Cell Signaling Technology Inc.), washed, and incubated for a further 1 h with 0.5 μg·mL^−1^ anti‐rabbit SULFO‐tag antibody. The plates were washed, 2 × Read Buffer T (Meso Scale Discovery) was added to wells, and electrochemiluminescence counts were made using a MSD SECTOR Imager 6000.

### 2.6 Immunoblotting

Western blotting of 20 μg of denatured lysates was carried out using precast 4–12% Bis/Tris gels in 1 × MOPS running buffer [Thermo Fisher Scientific (Invitrogen)]. A prestained molecular weight marker was loaded alongside the experimental samples (Invitrogen). The gels were transferred to PVDF membranes at 30 V for 3 h, or at 0.05 mA overnight. The membranes were blocked for 1 h in 5% (w/v) nonfat dry milk in Tris‐buffered saline with 0.1% Tween 20 (TBST), and then incubated with primary antibody overnight at 4 °C in 2.5% milk TBST (ALK, pY1278 ALK, pY1604 ALK, pY1586 ALK (all equivalent to the respective MSD^®^ immunoassay), ERK1/2, pERK1/2, Akt, pAkt, GAPDH, all from Cell Signaling Technology Inc.). Membranes were incubated with the appropriate horseradish peroxidase‐linked secondary antibody and proteins were visualized using electrochemical luminescence (ECL plus) [GE Healthcare Life Sciences (Amersham Biosciences), Buckinghamshire, UK] detection system on a LAS‐3000 Imaging System [GE Healthcare Life Sciences (Fujifilm), Buckinghamshire, UK]. Quantification of blots was performed by densitometry using imagej software analysis (Schneider *et al*., 2012).

### 2.7 Immunoprecipitation

Cell lysates were subjected to preclearing before direct incubation with the antibody of interest (ALK; IgG) for an hour at 4 °C. Protein G beads were added to the lysate and incubated overnight at 4 °C. The next day, the samples were spun down for 30 s at 4 °C and the supernatant was discarded. The bead pellet was washed five times with lysis buffer, before denaturing by heating to 95 °C for 5 min in SDS sample buffer.

### 2.8 Mouse models

All experiments, including the breeding of transgenic animals, were performed in accordance with the local ethical review panel, the UK Home Office Animals (Scientific procedures) Act 1986, the ARRIVE (Animal Research: Reporting of *In Vivo* Experiments) guidelines (Kilkenny *et al*., 2010) and the UK NCRI guideline (Workman *et al*., 2010). Th‐*ALK* ^*F1174L*^/*MYCN* tumor‐bearing animals were enrolled into therapeutic trials when their abdominal tumors reached 5 mm in diameter according to palpation. Volumetric MRI was performed as previously described (Jamin *et al*., 2013), with each animal undergoing imaging on day 0 and day 7. The tumor volume at each time point was then calculated. For *in vivo* oral dosing on days 1–7, crizotinib was dissolved in sterile water with 10% Tween 20. Ceritinib was dissolved in 0.5% methylcellulose, 0.5% Tween 80 with sterile water. Two hours following the final dose of either compound, tumor tissue was excised and snap‐frozen prior to analysis.

## 3 Results

### 3.1 Detection of recombinant ALK (rALK) protein with immunoassays

**Figure 1:**
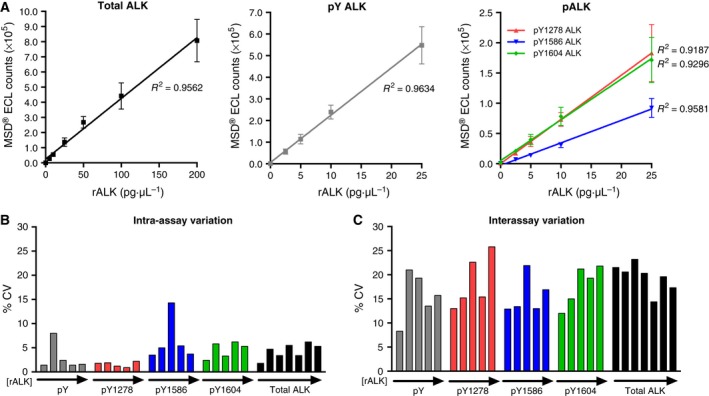
Immunoassays to quantitate phosphorylated and total ALK protein. (A) Titration of recombinant ALK protein (rALK) in MSD ^®^ assays to quantify pan‐phospho‐tyrosine ALK, phospho‐Y1278 ALK, phospho‐Y1586 ALK, phospho‐Y1604 ALK, and total ALK protein levels. Mean ± SD from ≥ 3 independent repeats. R‐squared values for linear regression from each of the assays are indicated. Assay reproducibility was assessed by (B) intra‐assay variability calculated across triplicate wells of each ALK MSD ^®^ assay with increasing concentrations of recombinant ALK protein (same as A) and (C) interassay variability calculated across from ≥ 3 independent repeats using increasing concentrations of recombinant ALK protein (same as A) and is presented as percentage coefficient of variation (CV).

Using the MSD^®^ platform, we optimized immunoassays to detect phosphorylated or total ALK protein. Confirmation of the ability of the assays to detect ALK or phosphorylated ALK species was sought through the use of a kinase active recombinant ALK (rALK) protein (Fig. 1). Using a titrating amount of rALK, we found that pan‐pY ALK, pY1278 ALK, pY1586 ALK, pY1604 ALK, and total ALK were detected in a linear fashion in each assay (Fig. 1A). The reproducibility of the assays to detect rALK was assessed both within a single experiment (Fig. 1B) and across at least three independent experiments (Fig. 1C). In all cases, the intra‐assay percentage coefficient of variation for pY1278, pY1604, and total ALK was less than 2.4%, 6.2%, and 6.2%, respectively. In four of five samples for the pY assay and the pY1586 assay, the coefficient of variation was less than 2.5% and 5.4%, respectively. The interassay variability was less than 30% in all assays, indicating high reproducibility of the assay signals.

### 3.2 Immunoassays quantitate ALK and phosphorylated ALK forms in neuroblastoma cell lines

**Figure 2:**
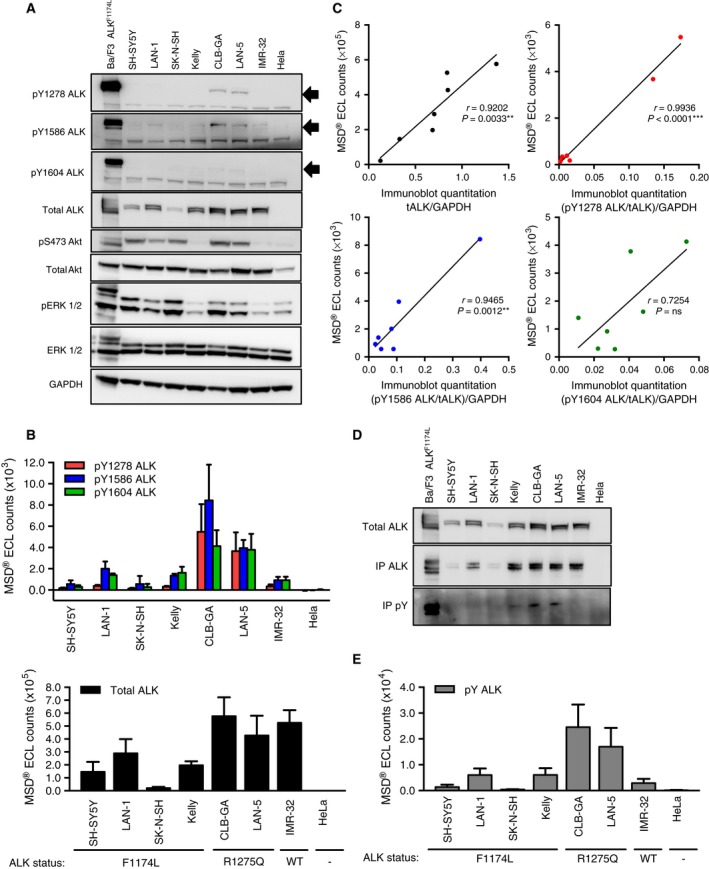
Basal ALK activity in neuroblastoma cell lines. (A) Immunoblots of lysates from neuroblastoma cell line panel, including lysate from Ba/F3 *ALK F1174L* as a positive control for ALK expression and lysate from Hela cells as negative control for ALK expression. Arrows indicate ALK band of interest at 220 kDa. (B) Quantitation of phospho‐Y1278 ALK, phospho‐Y1586 ALK, phospho‐Y1604 ALK, and total ALK protein levels in a cell line panel using immunoassays. Mean ± SD from ≥ 3 biological repeats. (C) Correlation of MSD ^®^ ECL counts with quantitation of total ALK (tALK) (*P* = 0.0033**) and phospho‐ALK (pY1278 ALK *P* = < 0.0001***; pY1586 ALK *P *= 0.0012**; pY1604 ALK *P* = ns (not significant)) immunoblots for neuroblastoma cell lysates. *r* values for Pearson's rank correlation of each graph are indicated. (D) Immunoprecipitation of total ALK from the same neuroblastoma cell line panel lysates (as in (A)), to allow the assessment of pan‐phospho‐tyrosine ALK status. (E) Quantitation of pan‐phospho‐tyrosine ALK, in a cell line panel by immunoassay. Mean ± SD from ≥ 3 biological repeats.

Gain‐of‐function mutations of *ALK*, which lead to constitutive ALK phosphorylation, are known to contribute to the aggressive nature of pediatric neuroblastoma tumors, and therefore, the detection of ALK activity in available neuroblastoma cell lines is of increasing importance. We employed a panel of these cell lines harboring either one of the two most frequent ALK mutations (F1174L or R1275Q), or wild‐type ALK, and assessed the total and phosphorylated abundance of ALK by immunoblotting, in addition to pathways downstream of ALK (Fig. 2A). When compared to the Ba/F3‐transduced cell lysates, it was apparent that detection and therefore quantification of phosphorylated ALK is challenging in the neuroblastoma cell panel by western blotting as bands were only clearly visible for pY1278 and pY1586 ALK in CLB‐GA and LAN‐5 lysates. Phosphorylated Akt and phosphorylated ERK 1/2 signals were detected more strongly in CLB‐GA and LAN‐5 again, as well as in three of the four *ALK F1174L* cell lines, excluding Kelly cells. However, analyzing the same cell lysates in the ALK immunoassays we developed showed detectable levels of pY1278, pY1586, pY1604, and total ALK in all the neuroblastoma cell lines tested, whereas no signals were obtained for HeLa cell lysate in any of the ALK assays consistent with the lack of ALK expression (Fig. 2B). When the levels of phosphorylated ALK in the neuroblastoma cell lines were normalized to total ALK signals from the immunoassays and compared with densitometry of the immunoblots, there was a positive correlation that was statistically significant for the total ALK and pY1278 and pY1586 ALK assays, and although not significant, a positive trend between densitometry and immunoassay for the detection of pY1604 ALK was seen (Fig. 2C). Furthermore, upon immunoprecipitation of ALK and detection of pan‐pY from the same lysates (Fig. 2D), there was a stronger pY signal obtained for the two cell lines harboring the R1275Q ALK mutation (CLB‐GA and LAN‐5). This finding is consistent with CLB‐GA and LAN‐5 cell lysates giving the highest signals out of the cell line panel in the pY1278, pY1586, and pY1604 ALK assays (Fig. 2B) and the pY ALK MSD^®^ assay (Fig. 2E). Together, these data indicate that the ALK immunoassays can be used to characterize basal levels of ALK activity in neuroblastoma cells in a more quantitative manner and with greater sensitivity than immunoblotting.

### 3.3 ALK immunoassays quantify the dose‐ and time‐dependent response of neuroblastoma cell lines to therapeutic ALK inhibition

**Figure 3:**
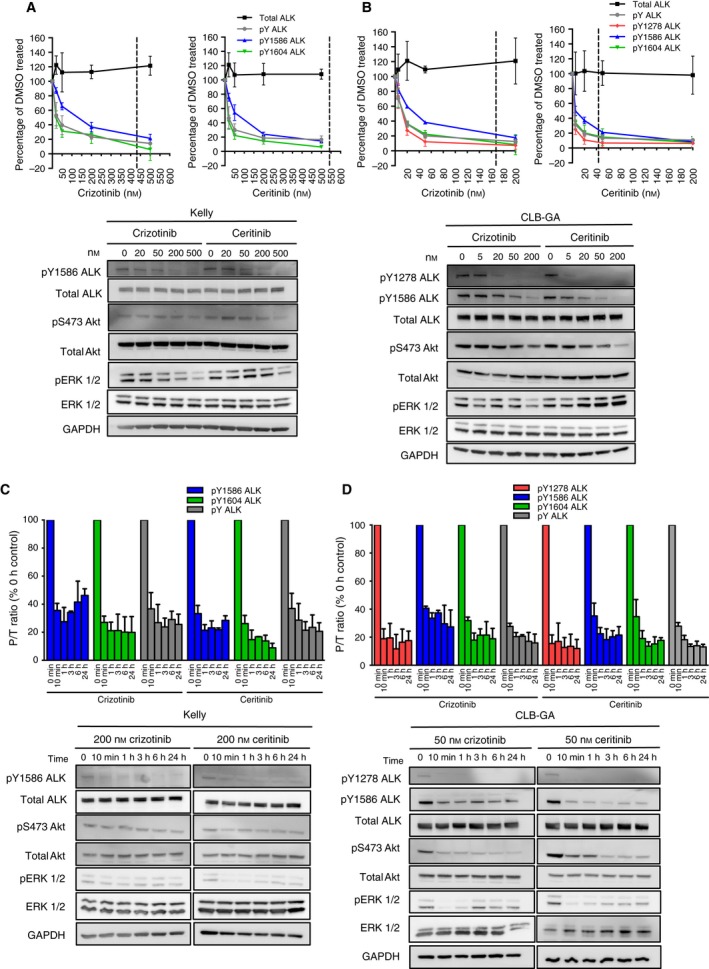
Dose–response and time course comparison of ALK inhibition by crizotinib or ceritinib. Dose response in Kelly (A) and CLB‐GA (B) neuroblastoma cells with 3‐hour treatment of crizotinib or ceritinib. Phosphorylated and total ALK levels were quantified by MSD ^®^ immunoassays, and mean ± SD from three independent repeats are shown. Corresponding immunoblots of Kelly and CLB‐GA cell lysates following the treatment with either crizotinib or ceritinib are shown below as indicated. Vertical dashed lines indicate GI_50_. Time course treatment of Kelly cells (C) with crizotinib or ceritinib (both 200 nm) and CLB‐GA cells (D) with crizotinib or ceritinib (both 50 nm). Phosphorylated and total ALK levels were quantified by MSD ^®^ immunoassays (three independent repeats), and the phosphorylated ALK/total ALK ratio as a % of untreated is plotted. Corresponding immunoblots of Kelly and CLB‐GA cell lysates following the treatment with either crizotinib or ceritinib are shown below as indicated.

To further assess the ability of the phospho‐ALK MSD assays to measure changes in ALK activity, we made use of two clinical small‐molecule ALK inhibitors, crizotinib and ceritinib. Crizotinib was less successful against ALK F1174L neuroblastomas in clinical trial, but showed efficacy in the treatment for ALK R1275Q neuroblastomas (Mosse *et al*., 2013). Ceritinib is a second‐generation ALK inhibitor, currently being evaluated in pediatric clinical studies. We used two neuroblastoma cell lines to compare crizotinib and ceritinib treatment in a dose‐dependent and time‐dependent manner using the total ALK, pY ALK, pY1278, pY1604, and pY1586 ALK immunoassays (Fig. 3). The drug concentrations were decided using the GI_50_ values for each compound (Table 1). Kelly cells (ALK F1174L) demonstrated a similar dose response to both drugs (Fig. 3A) with inhibition of ALK phosphorylation at sub‐GI_50_ concentrations, and this similarity was reflected in the accompanying immunoblot (pY1604 and pY1278 immunoblots did not detect bands and are therefore not included). CLB‐GA cells (ALK R1275Q) (Fig. 3B) were more sensitive to treatment with ceritinib than with crizotinib, with over 50% inhibition of phosphorylation seen with all phospho‐ALK assays at 10 nm ceritinib. This result is also seen in the accompanying immunoblot (pY1604 immunoblot did not detect bands and is therefore not included) (Fig. 3B). In both cell lines, loss in pY1586 ALK signal follows the pattern of decreasing pY ALK, pY1604 ALK, and pY1278 ALK (CLB‐GA only) with ALK inhibition and a reduction in band intensity in the accompanying immunoblots for pY1278 ALK. Although ALK phosphorylation was inhibited at sub‐GI_50_ concentrations of crizotinib or ceritinib, signaling pathways downstream of ALK, such as AKT and ERK1/2, were not inhibited until higher concentrations at 3‐hour treatment (immunoblots in Fig. 3A,B). The results of the dose–response experiments led to the choice of drug concentrations to be used in the subsequent time course experiments, as the dose that led to a percentage of DMSO treated at near 20% phosphorylated ALK.

**Table 1:** 72‐Hour GI_50_ of crizotinib or ceritinib in neuroblastoma cell lines (nm)

Cell lines	Crizotinib	Ceritinib
SH‐SY5Y	480.6 ± 93.0	82.0 ± 25.4
LAN‐1	236.6 ± 9.8	46.8 ± 36.3
SK‐N‐SH	3059.5 ± 272.2	1311.5 ± 252.4
Kelly	432.4 ± 28.6	542.4 ± 62.2
CLB‐GA	167.6 ± 48.3	43.4 ± 7.3
LAN‐5	132.0 ± 22.8	47.3 ± 6.3
IMR‐32	511.7 ± 29.1	1203.8 ± 386.7

Mean ± SD from three independent repeats.

There was a rapid decrease in the phosphorylated/total ALK ratio (P/T ratio) in Kelly cells (Fig. 3C) (graphed as percentage of zero‐hour control sample) following the treatment of the cells with either crizotinib or ceritinib. This effect was sustained for 24 h and is reflected in the accompanying immunoblots of the same lysates, where sustained inhibition of phosphorylated ERK1/2, a downstream effector of ALK, is also apparent following the treatment with either compound. Subtle inhibition of phosphorylation of Akt at S473 was also evident following the treatment of Kelly cells with crizotinib or ceritinib. Treatment of CLB‐GA cells (Fig. 3D) with either crizotinib or ceritinib also resulted in a rapid reduction in P/T ALK ratio from 10‐min treatment, an effect that was also sustained for the entire 24‐h time course. At 50 nm, there was some evidence that the downstream effectors of ALK were differentially affected following the treatment with either compound. Dephosphorylation of Akt at S473 occurred more rapidly after treatment of the cells with crizotinib; however, although treatment with either compound gave rise to a loss of ERK1/2 phosphorylation quickly, this effect was transient and not sustained for the duration of the experiment. Together, our results indicate that a sustained dephosphorylation of ALK at multiple phosphorylation sites by both crizotinib and ceritinib can be detected quantitatively by immunoassay in neuroblastoma cell lines with ALK mutations at F1174L or R1275Q.

### 3.4 Treatment of Th‐*ALK* ^*F1174L*^/*MYCN* tumor‐bearing animals demonstrates increased efficacy of ceritinib over crizotinib and a greater pharmacodynamic response

**Figure 4:**
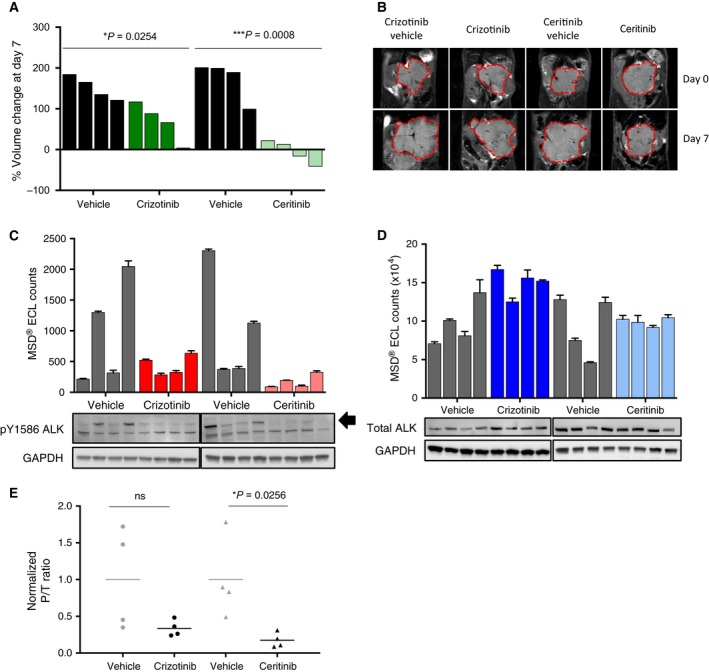
Tumor volume measurement and MSD ^®^ immunoassay quantitation of Th‐*ALK*^*F*^ ^*1174L*^/*MYCN* tumors following the treatment with crizotinib or ceritinib. Tumor‐bearing animals were treated with either crizotinib (100 mg·kg^−1^ per day, orally) or ceritinib (100 mg·kg^−1^ per day, orally) for 7 days before animal sacrifice and harvesting of tumor tissue samples. (A) MRI volumetric measurements were undertaken on day 0 and day 7, and percentage change in volume was calculated. (B) Representative abdominal MR images of matched coronal sections from individual animals included in the study on day 0 and day 7. Red dotted line indicates tumor. Quantitation of phospho‐Y1586 ALK (C) and total ALK (D) from Th‐*ALK*^*F*^ ^*1174L*^/*MYCN* tumor lysates using immunoassays. Mean ± SD from three technical repeats, with corresponding immunoblots shown directly underneath. Arrow indicates phospho‐Y1586 ALK band of interest at 220 kDa. (E) Phosphorylated ALK/total ALK ratios in tumor lysates calculated from immunoassay signals and normalized to mean of vehicle controls (normalized P/T ratio).

Finally, we tested frozen tissue specimens from Th‐*ALK* ^*F1174L*^/*MYCN* animals (in which spontaneous tumors arise, driven by coexpression of the human *ALK* ^*F1174L*^ and human *MYCN* transgenes), which had been treated with either crizotinib or ceritinib for 7 days. Treatment of animals with ceritinib (100 mg·kg^−1^ per day) results in delayed growth or tumor regression [established using noninvasive MRI volumetric measurements (Fig. 4B depicts representative images)], compared to delayed growth only following the treatment with crizotinib (100 mg·kg^−1^ per day) (Fig. 4A). The quantitation of pY1586 ALK and total ALK by immunoassay is shown next to the corresponding immunoblots (Fig. 4C,D). The ratio of pY1586 to total ALK revealed a mean decrease to 0.34 following crizotinib treatment versus 0.17 following ceritinib treatment (each normalized to the mean of their respective vehicle controls) (Fig. 4E), indicating a larger pharmacodynamic effect with ceritinib, consistent with the tumor volume data (Fig. 4A). Our results suggest that these immunoassays are suitable for the quantification of ALK as a biomarker following the targeted therapy *in vivo*.

## 4 Discussion

In this report, we have validated immunoassays to detect ALK and phosphorylated ALK *in vitro*, successfully applied them to an *in vivo* model of neuroblastoma, and demonstrated a quantitative difference in on‐target pharmacodynamic changes between a first‐ and second‐generation ALK inhibitor.

To date, the majority of studies investigating the mechanism of activation and phosphorylation of ALK utilize oncogenic ALK fusion proteins, such as NPM‐ALK. Activation of full‐length ALK has been shown to result in the phosphorylation of Y1278 alongside Y1282 and Y1283, which lie in the Y'Ras'YY autophosphorylation motif within the activation loop (Tartari *et al*., 2008). Y1604 is found at the C‐terminal tail of the receptor and has been reported to be important for transformation activity and docking of phospholipase Cy in studies on NPM‐ALK. Subsequently, Y1604 and Y1586 were together identified as tyrosine sites phosphorylated following ALK activation in a phosphoproteomics study of full‐length ALK (Sattu *et al*., 2013), and the detection of dephosphorylation at Y1586 by our immunoassay, following the treatment with ALK inhibitors, confirms the role of this phosphorylation site in the constitutive activity of ALK.

Our panel of neuroblastoma cell lines harbor either the *ALK F1174L* or the *ALK R1275Q* mutation or express wild‐type ALK only and therefore represent two of the three hotspots of ALK mutation sites in neuroblastoma tumors (Bresler *et al*., 2014). Our cross‐comparison of immunoblots with the ALK immunoassays reveals strong correlation of the detection of total ALK and two of three ALK phosphorylation sites (Fig. 2, Section 3.2). The immunoprecipitation of ALK and detection of pY furthermore reflect the results from the pY ALK immunoassay. Measurement of phosphorylated ALK by immunoassay has several advantages over western blotting such as greater quantitative power and higher‐throughput analysis of samples. The MSD^®^ platform we used also provides superior sensitivity and a much greater dynamic range in which to detect changes in ALK activity.

Interestingly, by analyzing a neuroblastoma cell line panel, we found that cells with the ALK R1275Q mutation had the strongest ALK phosphorylation signals. R1275 accounts for 43% of ALK mutations in neuroblastoma, yet mutations at the F1174 locus (accounting for 30% of ALK mutations) are reported to have the strongest effect on nonphosphorylated ALK tyrosine kinase domain *in vitro* (Bresler *et al*., 2014). It is possible that ALK activity is different in a whole‐cell environment and acclimatization to cell culture conditions alters the dominant cell signaling pathways and accounts for this discrepancy between purified mutated ALK tyrosine kinase domain experiments and our cell‐based approaches. Furthermore, we show that dephosphorylation at the pY1586 site closely followed dephosphorylation at the pY1278 and pY1604 autophosphorylation sites upon ALK inhibition, and therefore, phosphorylation of ALK at any of these three sites could be used as pharmacodynamic biomarkers for ALK activity.

Crizotinib and ceritinib are both under clinical evaluation for the treatment of ALK‐positive neuroblastomas with genetic ALK alterations, and our immunoassays allow for a direct pharmacodynamic preclinical comparison of the two compounds (Mosse *et al*., 2013). The observation that ceritinib has a lower GI_50_ in CLB‐GA than crizotinib is supported by the results of the immunoassay in these cells, where we see inhibition of ALK phosphorylation following the treatment with ceritinib at lower concentrations to crizotinib (Fig. 3, Section Acknowledgement). Downstream signaling mediators of ALK include the PI3K (phosphatidylinositol 3‐kinase)/Akt, the JAK/STAT (Janus activated kinase/signal transducer and activator of transcription), and Ras/Raf/MEK/ERK1/2 pathways (Berry *et al*., 2012; Sattu *et al*., 2013; Umapathy *et al*., 2014). The immunoblots show a return of signal for phosphorylated ERK1/2 after one hour of crizotinib treatment, but as the dephosphorylation of ALK at all phosphorylation sites remained evident for 24 h according to the immunoassay results, it could suggest that feedback mechanisms are activated or that cellular switching to up‐regulate alternative signaling pathways occurs. However, our immunoassays were able to specifically define the minimum concentration of inhibitor required to produce ALK dephosphorylation, and to demonstrate over what time period this occurs. The ability of our immunoassays to robustly quantify these ALK inhibitor characteristics in biological models will therefore provide valuable information to both their preclinical and clinical evaluation in defining active doses and optimum treatment schedules.

To assess the utility of the pY1586 ALK immunoassay to measure pharmacodynamic changes in an animal model treated with crizotinib or ceritinib, ALK phosphorylation status was quantified in *ex* *vivo* tumor tissue (Fig. 4, Section Author contributions). The transgenic Th‐*ALK* ^*F1174L*^/*MYCN* model develops spontaneous abdominal neuroblastomas in an immunocompetent background. The dephosphorylation of ALK detected by the immunoassay in this context by both compounds suggests that delivery of the drug results in on‐target inhibition of ALK in tumors. Moreover, the magnitude of dephosphorylation reflected the volumetric response of tumors to treatment with either inhibitor, and reinforced the conclusion that ceritinib is superior to crizotinib in the inhibition of ALK F1174L *in* *vivo*. The sensitivity and quantitative power of the immunoassay to detect these changes in phosphorylation in this context could be seen as a prelude for the future development of this assay as a tool to measure ALK activity as a clinical biomarker. In particular, we aim that our immunoassays could be embedded into pediatric clinical studies of ALK inhibitors in which biopsies at relapse are being encouraged. In this way, at the very least we would be able to correlate an observed clinical response to a targeted inhibitor with knowledge of the extent of ALK expression measured in the latest available biopsy. Additionally, these immunoassays will play a key role in the evaluation of ALK‐targeted agents in the panel of patient‐derived xenografts to be generated within the European Innovative Medicines Initiative Consortium.

## 5 Conclusions

Several inhibitors of the tyrosine kinase ALK have been evaluated in clinical studies for adult cancer patients, and the inherent resistance of the ALK mutations found in pediatric neuroblastomas necessitates thorough preclinical investigation of the available compounds. We have developed and validated quantitative immunoassays to measure total ALK and phosphorylated ALK at biologically relevant phosphorylation sites. These assays will support the preclinical decision‐making process in the evaluation of therapeutic ALK inhibitors using both *in vitro* and *in vivo* models.

## Author contributions

ERT and JRT performed the experiments and wrote the manuscript; LSD managed the transgenic colony; SG was consulted for the development of the immunoassay; transgenic MRI was undertaken by YJ under the leadership of SPR; UB and LC supervised the project.

## Funding

ERT was supported by a Medical Research Council and SPARKS Clinical Research Training Fellowship (MRC116X); JRT was supported by Abbie's Fund; LSD was supported by a Cancer Research UK Project Grant (A14619). YJ holds a Children with Cancer UK Research Fellowship.
